# Anti-HER2 treatment in everyday practice: how we treat older women with breast cancer differently

**DOI:** 10.1007/s10549-025-07888-z

**Published:** 2026-01-07

**Authors:** S. Hjorth, K. F. Vandraas, C. B. Trewin-Nybråten, E. Botteri, G. Ursin, B. K. Andreassen, N. C. Støer

**Affiliations:** 1 Department of Research, Cancer Registry of Norway, Norwegian Institute of Public Health, Oslo, Norway; 2 Norwegian Research Center for Women’s Health, Department of Obstetrics and Gynecology, Oslo University Hospital, Oslo, Norway; 3 Department of Oncology, Oslo University Hospital, Oslo, Norway; 4 Department of Registration, Cancer Registry of Norway, Norwegian Institute of Public Health, Oslo, Norway; 5 Section for Colorectal Cancer Screening, Cancer Registry of Norway, Norwegian Institute of Public Health, Oslo, Norway; 6 Cancer Registry of Norway, Norwegian Institute of Public Health, Oslo, Norway; 7 Institute of Basic Medical Sciences, University of Oslo, Oslo, Norway; 8 Department of Preventive Medicine, University of Southern California, Los Angeles, CA USA

**Keywords:** HER2-positive, Breast cancer, Older patients, Anti-HER2 targeted therapies

## Abstract

**Purpose:**

Targeted therapies have improved survival in human epidermal growth factor receptor 2 positive breast cancer (HER2 + BC). However, patients over 75 years of age are often excluded from clinical trials of anti-HER2 therapies, and it is unclear to what extent they receive these treatments in routine care. To address this, we examined age-related patterns of anti-HER2 therapy use in real-world clinical practice in Norway.

**Methods:**

In a nationwide registry-based cohort, we identified women diagnosed with stage I-III HER2 + BC during 2012–2021. We investigated treatment patterns using descriptive statistics and estimated the direct effect of age on anti-HER2 therapy use by Poisson regression.

**Results:**

Among 3526 women with HER2 + BC, anti-HER2 therapy use was consistently high (83–95%) in those under 75 years, decreased to 60% at ages 75–79, and declined further with advancing age to 8% at ≥ 90 years. Neoadjuvant anti-HER2 therapy also decreased with age (from 24% in patients under 75 to 12% in patients over 75 years). Accounting for cancer characteristics, comorbidities, polypharmacy, and socio-economic factors, older patients had reduced likelihood of receiving any anti-HER2 therapy compared with patients younger than 55 (RR 0.75, 95% CI 0.66–0.85, p < 0.001, at age 75–84 and RR 0.21, 95% CI 0.11–0.41, p < 0.001, at age 85 +).

**Conclusions:**

Anti-HER2 therapy use declined substantially after the age of 75 even when accounting for comorbidities and polypharmacy. Chronological age appears important in planning treatment for patients with HER2 + BC. Specific guidelines pertaining to older patients with HER2 + BC are needed to avoid potential undertreatment.

**Supplementary Information:**

The online version contains supplementary material available at 10.1007/s10549-025-07888-z.

## Introduction

Older patients, especially those over 75 years, are under-represented in pivotal clinical trials leading to cancer therapy approval and implementation in clinical practice [1]. This represents a challenge for clinicians when planning treatment for older patients. Historically, patient’s age has had a decisive impact on treatment guidelines. During later years, consensus statements recommend comprehensive geriatric assessment (CGA) of patient frailty before treatment initiation, not relying solely on age [2]. Yet studies indicate that less than half of breast cancer (BC) patients over 70 years receive a CGA prior to treatment initiation [3–5].

Approximately 15% of BC patients present with HER2 + disease (HER2 + BC), a prognostic trait that guides adjuvant treatment planning [6]. At present, adjuvant therapy for HER2 + BC routinely includes one year of targeted anti-HER2 therapy, combined with chemotherapy for three or six months [7]. Neoadjuvant therapy with dual anti-HER2 therapy (trastuzumab and pertuzumab) and chemotherapy is recommended for patients with larger tumours (> T2) or locally advanced disease [7]. Based on individual assessments, anti-HER2 therapy may be omitted for patients presenting with small tumours and no lymph node metastasis (eg pT1aN0) [7, 8].

Among patients with HER2 + BC, older age has been shown to be associated with reduced use of trastuzumab in combination with chemotherapy [9]. This is unsurprising, given the risk of toxicity in frail patients treated with chemotherapy [10]. However, trastuzumab monotherapy is generally well-tolerated [10] and recommended for use also in older patients [2]. Thus, it is important to study the impact of age on targeted anti-HER2 therapy decisions independent of chemotherapy. One previous study did so among 46 patients with HER2 + BC and found that 67% of those considered “fit” after CGA and 58% of those considered “unfit” received anti-HER2 targeted therapy [11]. This suggests that factors other than patient frailty influence anti-HER2 treatment planning. While one factor could be chronological age, low-income status has also been shown to reduce the likelihood of receiving treatment among older BC patients [12]. By examining treatment patterns among older patients within a health care system where cancer care is publicly funded and private treatment is uncommon [13], we can generate insights that may help safeguard the quality of cancer care in older patients.

We took advantage of population-based registries to describe curative treatment of HER2 + BC across a 10-year period, aiming to explore treatment patterns by age in modern oncological care. We also assessed the impact of chronological age on the likelihood of receiving anti-HER2 therapy while accounting for disease characteristics and patient health.

## Methods

This registry-based cohort included all women 18 years or older diagnosed with HER2 + BC in Norway from 2012 to 2021. During this period, Norwegian treatment guidelines changed. Until mid-2013, systemic adjuvant treatment of BC patients over 75 years was not routinely recommended. Since then, guidelines have recommended individual assessments [14] consistent with international guidelines [2].

Using the unique personal identification number given to all residents in Norway, we linked information from the Cancer Registry of Norway (CRN), the Norwegian Patient Registry (NPR), the Norwegian Prescribed Drug Registry (NorPD), Statistics Norway and the Norwegian Cause of Death Registry. All registries are based on mandatory notification.

### Data sources

The CRN has recorded incident cancer cases since 1952. It is considered practically complete for solid tumours outside the central nervous system [15]. The CRN, including the incidence database [16] and the Norwegian Breast Cancer Registry [17], provided information on patient age at diagnosis, tumour characteristics, surgery, and date of metastasis. The CRN also obtains information on systemic anti-cancer treatment (SACT) [18], which includes information on all antineoplastic drugs (Anatomical Therapeutic Chemical (ATC) group L01 [19]) administered in Norwegian public hospitals except for the northern health region. The coverage is 91% for 2019–2021, but some information is available for earlier years as well.

The NPR has collected individual level data from in- and outpatient hospital encounters in all public hospitals in Norway since 2008 [20]. From the NPR, we received information on antineoplastic treatment for the years and health region not covered by the CRN’s SACT collection. We also used information on metastatic diagnoses (International Classification of Diseases 10th Revision (ICD-10) codes C77-79 [21]) to supplement information from the CRN. Finally, we gathered information on comorbidities.

The NorPD was established in 2004 [22]. It records information on all prescriptions redeemed at pharmacies by patients in outpatient care. We used NorPD to obtain information on cancer treatments (ATC groups L01 and L02) used in the outpatient setting and on polypharmacy.

From Statistics Norway we received information on educational level and household income, and from the Norwegian Cause of Death Registry [23], we obtained information on time and cause of death.

### Study population

Of 4696 patients diagnosed with HER2 + BC (ICD-10 code C50) from 2012 to 2021, we excluded 272 patients with de novo metastatic disease (stage IV at diagnosis or within 4 months after diagnosis) (Supplementary Figure S1). A further 221 patients were excluded due to missing information on cTNM stage at diagnosis. Among the remaining 4203 patients diagnosed at stage I–III, we excluded patients with non-carcinomas (n < 5), previous cancer (apart from non-melanoma skin cancer) (n = 418), or another cancer (apart from breast or non-melanoma skin cancer) diagnosed on the same day as their BC (n < 5). Patients with more than one BC diagnosis on the same day were included and classified according to the most aggressive histology registered.

In the NPR, some cancer treatments were only registered with an unspecific treatment code (“medical treatment against tumours”). We attempted to link unspecific treatment codes to a known treatment based on a modified algorithm developed by Sørup et al. [24] (see Supplementary methods). If 48 days or less passed between a specific and an unspecific treatment code, they were defined as belonging to the same treatment line. If the unspecific treatment occurred with more than 48 days to another treatment, it remained unspecified. Patients who had one or more unspecified treatments after application of the algorithm were excluded (n = 252), yielding a final study sample of 3526 patients.

The patients were followed from cancer diagnosis until death, other cancer diagnosis, or June 2023, whichever came first. Treatment information was available to 2022 (exact end date differed by registry) and was censored at metastatic progression.

### Variables

Patient age at diagnosis was categorized as < 55 years, 55–64 years, 65–74 years, 75–84 years, and 85 + years. Narrower (5-year intervals) and broader (< 55, 55–74 and 75 +) categories were used when needed.

Treatment was categorized as anti-HER2 -, chemo-, and endocrine therapy (for ATC-codes, see Supplementary methods). During the study period, the available anti-HER2 therapies in the curative setting were trastuzumab monotherapy, trastuzumab and pertuzumab combination therapy, and trastuzumab emtansine. The chemotherapies used in the curative setting were taxanes, anthracyclines in combination with cyclophosphamide, antimetabolites, platinum compounds, vinorelbine and eribulin.

Neoadjuvant treatment was defined as receiving any systemic therapy from ATC groups L01 or L02 in the 18 months before curative surgery, though not preceding the incident BC diagnosis date. Adjuvant treatment was defined as anti-HER2 therapy within 18 months following surgery, endocrine therapy within 12 months following surgery, taxanes or anthracyclines within 6 months following surgery, and other chemotherapy within 12 months after surgery. The intervals were chosen to allow grace periods and capture all relevant treatment. Curative treatment was defined as neoadjuvant or adjuvant treatment.

We calculated a previously validated comorbidity index at the time of diagnosis based on ICD-10 codes reported by the hospitals to the NPR in the four years before incident BC [25]. Values above zero reflected in- or outpatient hospital admission for one or more comorbidities. The comorbidities included in the index are the same as for the Charlson Comorbidity Index, but they are weighted differently in adaption to a Norwegian setting.

To further assess general health, we identified patients with polypharmacy from NorPD, defined as redeeming 8 or more different medications (different ATC-codes) from the pharmacy in the year before BC diagnosis. This definition has been used in previous studies [26]. We also identified the number of hospital contacts in the year before diagnosis from NPR as an indirect measure of health care utilisation.

### Statistical methods

#### Patient characteristics were presented as medians and percentages

We investigated the proportion of patients using any anti-HER2 therapy, chemotherapy, and endocrine therapy (among hormone receptor positive patients) according to age group, as well as number of adjuvant anti-HER2 therapy cycles and type of chemotherapy. For this analysis, age was categorized in 5-year age groups with the upper and lower age interval determined to ensure at least 10 patients per group. This was done to more accurately show at what age treatment patterns change, while retaining adequate sample size. As BC stage at diagnosis varies with patient age due to national BC screening strategies, we did a sensitivity analysis stratified by clinical stage. In a second sensitivity analysis attempting to disentangle age and comorbidity, we restricted the analysis to patients with a comorbidity index of zero and no polypharmacy. We also performed a sensitivity analysis restricted to patients diagnosed during 2015–2021 to exclude patients diagnosed at a time when Norwegian guidelines did not routinely recommend systemic adjuvant treatment of BC patients over 75 years.

We displayed patient flows including neoadjuvant anti-HER2 therapy, surgery, adjuvant and post-neoadjuvant anti-HER2 therapy by age group using a Sankey diagram [27].

Finally, we explored patient characteristics by age group according to use or non-use of any anti-HER2 therapy (ATC-group L01FD). As some patients might not have been followed long enough to commence treatment, this analysis excluded patients with shorter follow-up than 56 days from diagnosis, which corresponds to the maximum time from surgery to treatment allowed in the APHINITY trial that compared adjuvant combination therapy with trastuzumab and pertuzumab to trastuzumab monotherapy [28]. The 56 days are also in line with Norwegian national health care standards for treatment initiation (10 days from diagnosis to neoadjuvant and 55 days to adjuvant treatment start) [29].

Among patients with at least 56 days follow-up, we also estimated the direct effect of age on anti-HER2 therapy use through adjustment for potential mediators of the age-treatment association (see Supplementary methods) [30]. To do this, we used a Poisson regression with robust standard errors [31] adjusted for cancer characteristics (calendar year, stage, and grade at diagnosis), socioeconomic factors (household income, education, and region of residence) and general health (comorbidity index, polypharmacy, and number of hospital contacts). Covariates were categorized as in Table 1 except for year of diagnosis, which was included as a continuous variable. In 11% of the sample, data were missing on one or more covariates and were imputed using multiple imputation (see Supplementary methods) [32]. Post hoc, we decided to perform the crude and adjusted regression analysis restricted to patients diagnosed in 2015–2021 with indication for anti-HER2 therapy (stage I tumour > pT1a or stage II-III), a comorbidity index of zero and no polypharmacy. The rationale for this was to eliminate observed interactions between age and diagnosis year, age and comorbidity, and age and polypharmacy.

**Table 1:** Patient characteristics by age group among 3526 Norwegian women with primary non-metastatic HER2 positive breast cancer

	Total	< 55 years	55–64 years	65–74 years	75–84 years	85 + years
n	3526	1628	824	602	324	148
Sociodemographics						
Year of diagnosis						
2012–2014	1058 (30.0%)	474 (29.1%)	261 (31.7%)	173 (28.7%)	96 (29.6%)	54 (36.5%)
2015–2021	2468 (70.0%)	1154 (70.9%)	563 (68.3%)	429 (71.3%)	228 (70.4%)	94 (63.5%)
Health region of residence at diagnosis						
Southeast	2070 (59.0%)	970 (60.0%)	465 (56.8%)	387 (64.5%)	176 (54.3%)	72 (48.6%)
West	587 (16.7%)	276 (17.1%)	139 (17.0%)	73 (12.2%)	60 (18.5%)	39 (26.4%)
Mid	525 (15.0%)	229 (14.2%)	130 (15.9%)	85 (14.2%)	54 (16.7%)	27 (18.2%)
North	326 (9.3%)	142 (8.8%)	85 (10.4%)	55 (9.2%)	34 (10.5%)	10 (6.8%)
Missing	18	11	5	(n < 5)	0	0
Educational attainment						
Compulsary	679 (19.5%)	235 (14.7%)	161 (19.8%)	120 (20.1%)	103 (32.3%)	60 (40.8%)
Secondary	1462 (42.0%)	554 (34.5%)	377 (46.4%)	316 (52.9%)	151 (47.3%)	64 (43.5%)
Higher	1338 (38.5%)	815 (50.8%)	274 (33.7%)	161 (27.0%)	65 (20.4%)	23 (15.6%)
Missing	47	24	12	5	5	(n < 5)
Household income according to national quintiles						
Lowest quintile	729 (20.8%)	183 (11.3%)	151 (18.4%)	145 (24.2%)	147 (45.5%)	103 (69.6%)
Middle 3 quintiles	2068 (59.0%)	964 (59.7%)	494 (60.2%)	400 (66.7%)	169 (52.3%)	41 (27.7%)
Highest quintile	709 (20.2%)	467 (28.9%)	176 (21.4%)	55 (9.2%)	7 (2.2%)	(n < 5)
Missing	20	14	(n < 5)	(n < 5)	(n < 5)	0
General health						
Comorbidity index > 0	385 (10.9%)	71 (4.4%)	63 (7.6%)	117 (19.4%)	86 (26.5%)	48 (32.4%)
Polypharmacy	817 (23.2%)	188 (11.5%)	175 (21.2%)	219 (36.4%)	161 (49.7%)	74 (50.0%)
Number of hospital contacts						
0	3134 (88.9%)	1554 (95.5%)	762 (92.5%)	473 (78.6%)	239 (73.8%)	106 (71.6%)
1–2	240 (6.8%)	46 (2.8%)	43 (5.2%)	74 (12.3%)	51 (15.7%)	26 (17.6%)
3 +	152 (4.3%)	28 (1.7%)	19 (2.3%)	55 (9.1%)	34 (10.5%)	16 (10.8%)
Tumour characteristics						
Hormone receptor positive	2414 (68.6%)	1139 (70.1%)	552 (67.2%)	411 (68.5%)	218 (67.3%)	94 (63.5%)
Missing	8	(n < 5)	(n < 5)	(n < 5)	0	0
Histological grade						
1	104 (3.3%)	47 (3.2%)	24 (3.1%)	18 (3.1%)	10 (3.5%)	5 (4.4%)
2	1209 (37.8%)	527 (36.2%)	310 (40.2%)	240 (42.0%)	89 (31.3%)	43 (37.7%)
3	1883 (58.9%)	880 (60.5%)	438 (56.7%)	314 (54.9%)	185 (65.1%)	66 (57.9%)
Missing	330	174	52	30	40	34
Clinical stage						
I	1272 (36.1%)	529 (32.5%)	355 (43.1%)	277 (46.0%)	90 (27.8%)	21 (14.2%)
II	1567 (44.4%)	745 (45.8%)	343 (41.6%)	234 (38.9%)	157 (48.5%)	88 (59.5%)
III	687 (19.5%)	354 (21.7%)	126 (15.3%)	91 (15.1%)	77 (23.8%)	39 (26.4%)

Since not all patients in our cohort underwent surgery, we could not base exclusion on 56 days of follow-up from surgery. We therefore performed a sensitivity analysis excluding all patients with shorter follow-up from diagnosis than the 95th percentile for ‘time from diagnosis to anti-HER2 therapy’ among treated patients. The same cut-offs were used regardless of surgery status.

All analyses were performed in Stata (Stata Corp. 2023. Stata Statistical Software: Release 18. College Station, TX: Stata Corp LLC).

## Results

Among 3526 patients diagnosed with stage I–III HER2 + BC in Norway from 2012 to 2021, 472 (13%) were 75 years or older (Table 1). Older patients had lower education and household income, and a higher prevalence of comorbidities at diagnosis compared to younger patients.

**Fig. 1:**
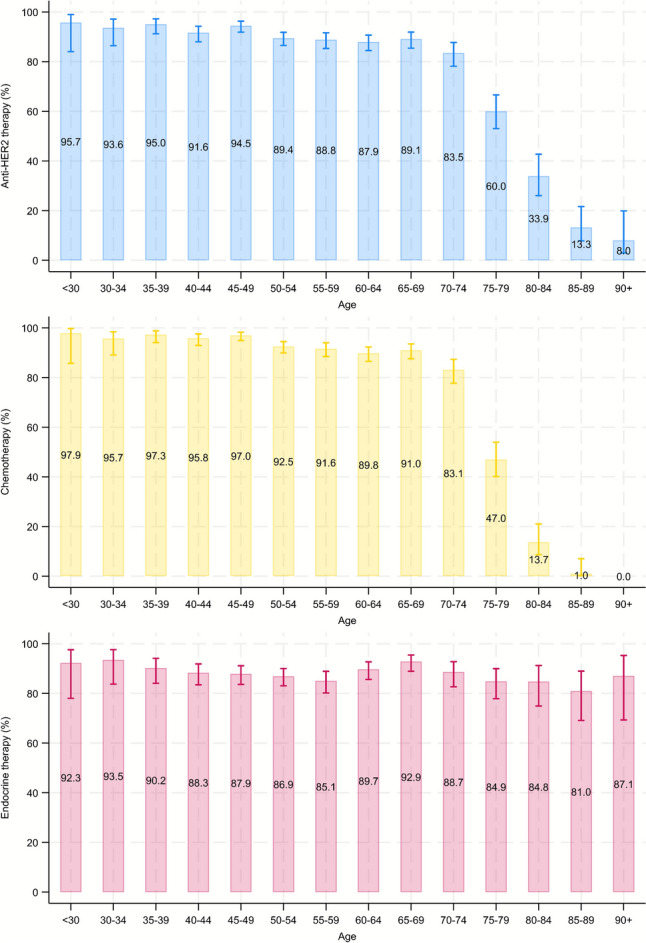
Proportion of systemic treatment use by age group among 3526 Norwegian women with primary non-metastatic HER2 positive breast cancer. For endocrine therapy, the denominator is patients with hormone receptor positive disease

The proportion of patients receiving anti-HER2 therapy declined after age 75 from 83% at age 70–74 to 60% at age 75–79 and 8% at age 90 + (Fig. 1). The median number of adjuvant treatment cycles among treated patients was 17 cycles before age 80, 16 cycles at age 80–84, and 5 cycles at age 85–89. No patients over 90 years received anti-HER2 therapy after surgery. Chemotherapy use declined even more with increasing patient age, from 83% at age 70–74, to 47% at age 75–79 and 0% at age 90 + . Among those who received chemotherapy, the proportion receiving taxanes remained stable across age, whereas the proportions receiving anthracyclines and antimetabolites declined after age 75 (Supplementary Table 1). This contrasted with endocrine therapy among hormone receptor positive patients, where we observed no clear age-differences. The sensitivity analyses stratified on stage (Supplementary Figure S4) resembled the main analysis for stages I and II, whereas the decline in anti-HER2 therapy use after age 75 among stage III patients was less pronounced (73% of patients aged 75–79 compared to 81% of patients aged 70–74). Restriction to patients with a comorbidity index of zero and no polypharmacy (Supplementary Figure S5), and restriction to patients diagnosed in 2015–2021 (Supplementary Figure S6), showed higher proportions of anti-HER2 therapy use overall, yet declines in therapy use after age 75 were similar to the main analysis.

**Fig. 2:**
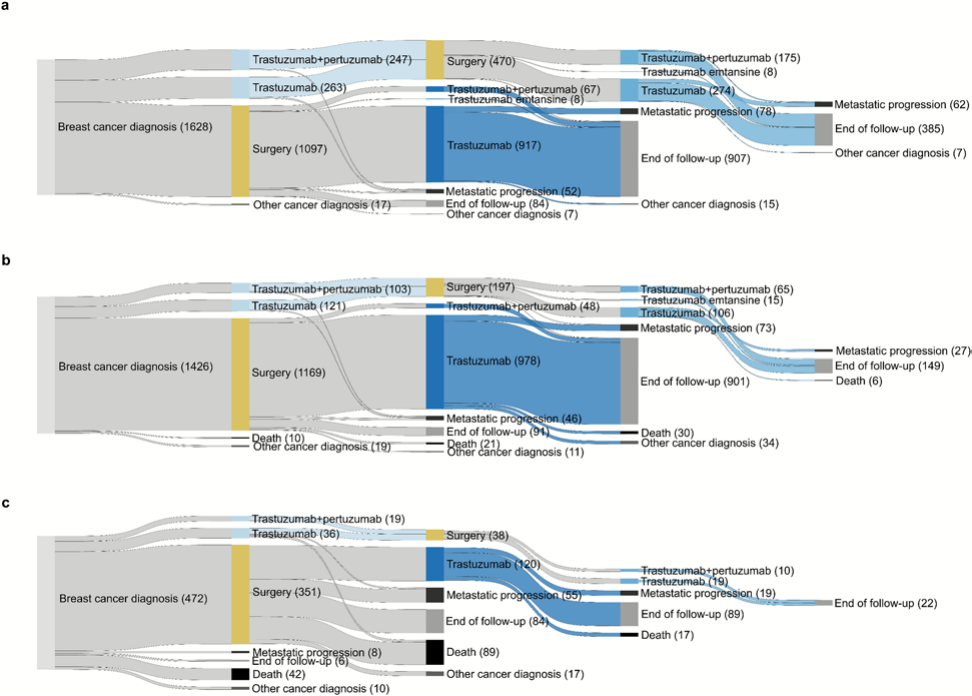
Treatment flow (neoadjuvant treatment, surgery and first line adjuvant treatment) by age group among 3526 Norwegian women with primary non-metastatic HER2 positive breast cancer. Panel **a** patients aged < 55 years; panel **b** patients aged 55–74 years; panel **c** patients aged 75 + years. Light blue colour represents neoadjuvant, dark blue adjuvant, and middle blue post-neoadjuvant treatment. Paths with < 5 patients are not shown

The youngest patients received neoadjuvant therapy more often than other age groups (31% in patients aged < 55 years, 16% in patients aged 55–74, and 12% in patients aged 75 +) (Fig. 2, Supplementary Table 2). The proportion of patients receiving trastuzumab in combination with pertuzumab in the adjuvant setting declined with age from 7% in patients < 55 years, 5% at age 55–74, to < 4% (< 5 patients) at age 75 + . Most (65%) primary surgically treated patients aged 75 + either progressed to metastasis, were censored, reached end of follow-up or died before receiving adjuvant anti-HER2 therapy compared with 10% at age < 55 years and 12% at age 55–75.

**Fig. 3:**
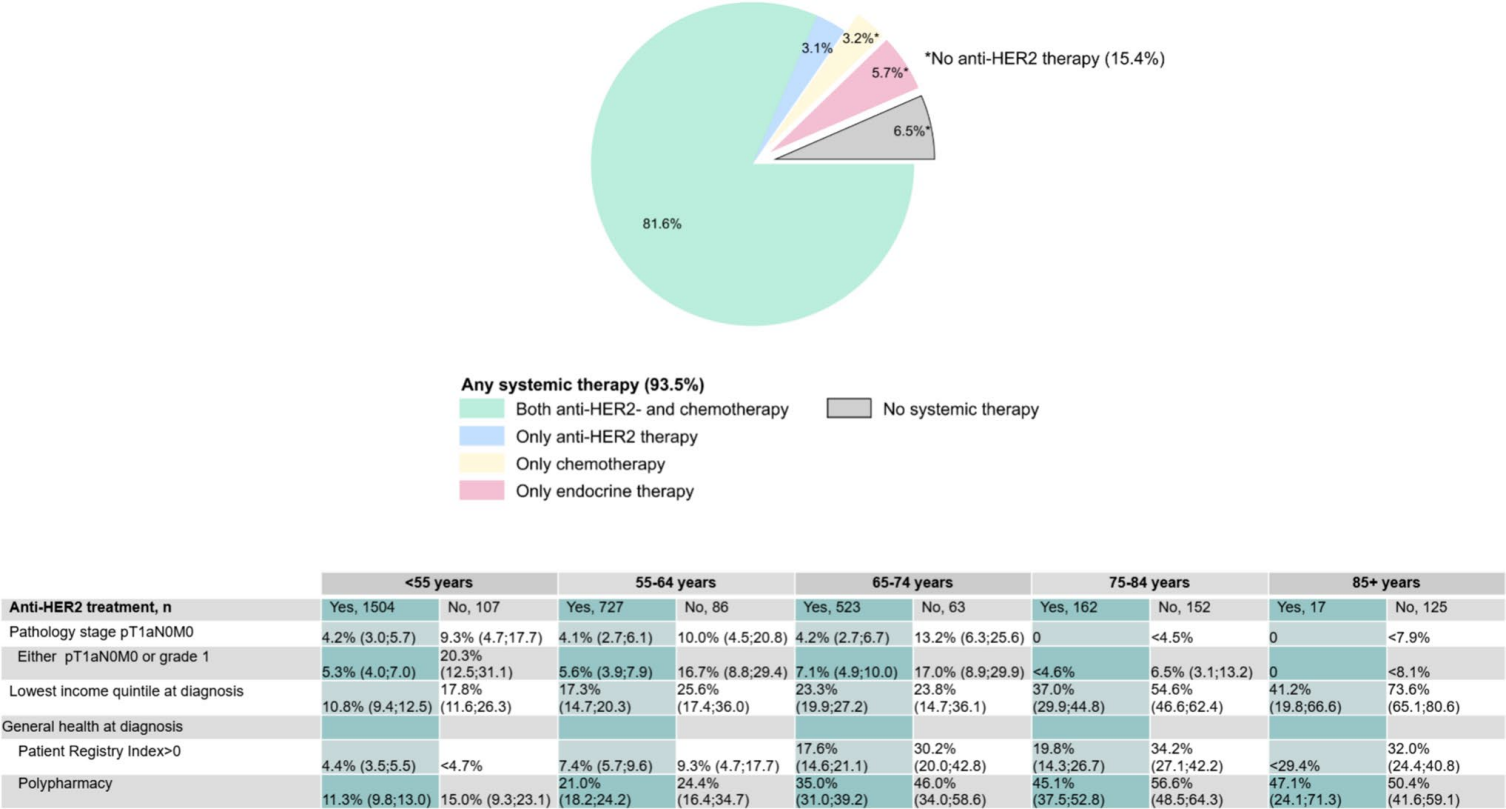
Characteristics of patients receiving and not receiving systemic antineoplastic treatment (Anatomical Therapeutic Chemical group L01) by age group among 3466 Norwegian women with primary non-metastatic HER2 positive breast cancer who had at least 56 days of follow-up. The percentages shown, are percentages of non-missing values. Exact percentages are not shown when < 5 patients in a category

Among 3466 patients with 56 days or more of follow-up, 2933 (85%) received anti-HER2 therapy, 307 (9%) received chemotherapy or endocrine therapy only, and 226 (7%) did not receive any systemic therapy (Fig. 3). Compared to patients who received anti-HER2 therapy, patients who did not receive anti-HER2 therapy more often had comorbidity and low income at diagnosis. However, these differences were most pronounced for the oldest patients.

Upon adjustment for cancer characteristics, socioeconomic factors and patient health, we still observed a lower likelihood of receiving anti-HER2 therapy among older patients. Indeed, adjustment affected the estimates of association very little (Table 2). The likelihood of receiving treatment was not different in patients aged 55–64 (RR 0.97, 95% CI 0.95–1.00) or 65–74 (RR 0.99, 95% CI 0.96–1.02) compared to < 55 years, whereas the likelihood of receiving treatment was much lower in patients aged 75–84 years (RR 0.59, 95% CI 0.53–0.65) and 85 + (RR 0.14, 95% CI 0.09–0.22). Among patients diagnosed in 2015–2021 with indication for anti-HER2 therapy, a comorbidity index of zero and no polypharmacy, the RR of receiving anti-HER2 therapy was attenuated to 0.75 (95% CI 0.66–0.85) at age 75–84 and 0.21 (95% CI 0.11–0.41) at age 85 + . Sensitivity analyses were comparable to the main analysis (Supplementary Figure S7 and Supplementary Table 3).

**Table 2:** Association between age and use of anti-HER2 targeted therapy among 3466 Norwegian women with primary non-metastatic HER2 positive breast cancer with at least 56 days of follow-up

	n	Anti-HER2 (%)	Crude RR (95% CI)	Adjusted^a^ RR (95% CI)
Full imputed sample				
< 55	1611	1504 (93.4)	Reference	Reference
55–64	813	727 (89.4)	0.96 (0.93–0.98)	0.97 (0.95–1.00)
65–74	586	523 (89.2)	0.96 (0.93–0.99)	0.99 (0.96–1.02)
75–84	314	162 (51.6)	0.55 (0.50–0.62)	0.59 (0.53–0.65)
85 +	142	17 (12.0)	0.13 (0.08–0.20)	0.14 (0.09–0.22)
Patients diagnosed in 2015–2021 who had tumour stage I > pT1a, or II–III, PRI = 0 and no polypharmacy, imputed sample				
< 55	916	879 (96.0)	Reference	Reference
55–64	384	356 (92.7)	0.97 (0.94–1.00)	0.97 (0.94–1.00)
65–74	208	190 (91.3)	0.95 (0.91–0.99)	0.95 (0.91–1.00)
75–84	91	65 (71.5)	0.75 (0.65–0.85)	0.75 (0.66–0.85)
85 +	35	7 (20.0)	0.21 (0.11–0.40)	0.21 (0.11–0.41)

^a^Adjusted for calendar year at diagnosis, stage, grade, household income, education, comorbidity index, polypharmacy and number of hospital contacts. In the restricted sample, comorbidity index and polypharmacy are not included in the adjustment model

*RR* relative risk, *CI* confidence interval, *PRI* Patient Registry Index, a Norwegian validated comorbidity index

## Discussion

In this population-based cohort of patients with early-stage HER2 + BC, patients older than 75 years were less likely than younger patients to receive anti-HER2 therapies both in the neoadjuvant and adjuvant setting. This finding remained after accounting for disease characteristics, patient comorbidities, and polypharmacy.

There is substantial evidence for the benefit of CGA when planning oncological treatment among older patients [33]. However, our results indicated that chronological age influenced anti-HER2 treatment decisions even after we accounted for cancer characteristics, socioeconomic factors, and patient health. This is in line with a previous study, where age, but not CGA result, predicted chemotherapy use among BC patients over 70 years [3].

We also observed an association, albeit attenuated, between older age and reduced likelihood of receiving anti-HER2 therapy for patients diagnosed in 2015–2021. At this time guidelines emphasised individual assessment of treatment eligibility, not age alone, especially for patients with HER2 + disease [14]. This suggests that it takes time for changes in the guidelines to become integrated in clinical practice.

We were surprised to observe similar declines in the usage of both anti-HER2 therapy and chemotherapy in older age groups, because tolerability for anti-HER2 therapies is considered better than for chemotherapy, and anti-HER2 therapies are recommended also in older age groups [14]. Our findings highlight the need for dedicated treatment guidelines for geriatric BC patients. Such guidelines could facilitate more appropriate treatment selection in the older population and increase awareness of trastuzumab monotherapy as a viable option for patients unable to tolerate chemotherapy [2].

The decision to refrain from anti-HER2 therapy in the oldest age group, even among seemingly healthy patients, may be due to lack of evidence from clinical trials on effect and safety for older patients [34]. Furthermore, oncologists may weight perceived benefits of treatment against the risk of adverse effects and patients’ remaining life-expectancy [34]. Indeed, among patients aged 85 + the median number of anti-HER2 treatment cycles was only 5, suggesting that those who did receive anti-HER2 therapy were not able to complete the full 17 treatment cycles. However, this was not the case for patients younger than 85 years.

Other potential explanations for the decline in systemic therapy with age could be problems with access to treatment (eg reduced mobility or dependency upon family members) and patient preferences; information we unfortunately did not have. While we have not identified any studies on anti-HER2 therapy preferences in older patients, one study found older early-stage BC patients more likely to refuse endocrine—and chemotherapy than younger patients [35]. Older age is also associated with patients valuing quality of life above length of life when making treatment decisions for early-stage BC [36].

Patients who did not receive anti-HER2 therapy had lower household income than treated patients, especially in the older age groups. This finding is in line with studies from other settings [12], but unexpected, given that all cancer treatment in Norway is free of charge. However, household income depends on education and employment (both highly correlated to age), and the number of people in the household. As such, low income may indicate social vulnerability or widowhood and capture other aspects of patient frailty than comorbidity.

### Strengths and limitations

A major strength of this study is the population-based design, including all primary incident early stage HER2 + BC in Norway during a decade, thus reducing the risk of selection bias. We obtained comprehensive treatment data from population-based registries based on mandatory reporting. This ensured high completeness and allowed us to consider not only in-hospital -, but also outpatient treatments.

The most important limitation of this study is that we did not have access to individual patient medical records. We were therefore not able to assess the physician’s rationale for non-treatment in each individual case, including whether a CGA was performed. While we had access to information on comorbidities, we did not have information about the patients’ functional status, for instance as measured by the Eastern Cooperative Oncology Group Performance Status Scale.

Furthermore, we did not have information about medications administered in nursing homes. This could result in an increasing underestimation of polypharmacy with increasing age. However, the underestimation is unlikely to be substantial, as endocrine therapy use did not decrease with age though primarily administered in the outpatient setting. The potential for misclassification of anti-HER2 therapies is low, as none of the relevant drugs are administered in nursing homes.

Finally, we did not have access to any treatments that patients might have received abroad or in private hospitals. However, as cancer care is free of charge in public hospitals, very few patients in Norway receive cancer therapy in private hospitals (around 100 per year across all cancer sites) [13]. Seeing that untreated patients had lower income than those treated, it seems unlikely that a substantial proportion of patients were misclassified as untreated while receiving private sector care. We cannot rule out that some, most likely younger patients, received therapies in private hospitals while awaiting national approval. This applies to pertuzumab during 2013–2017 in the neoadjuvant, and 2017–2019 in the adjuvant setting, and T-DM1 during 2019–2020. However, these patients would probably shift to public health care, registered in the NPR, upon drug approval. Further, patients receiving pertuzumab in private care would likely receive approved drugs (trastuzumab and chemotherapy) in the public hospitals. As such, they would not be classified as untreated in our study.

## Conclusions

Our findings suggest that patients over 75 years with HER2 + BC are vulnerable to undertreatment in the curative setting. Chronological age appears to play a decisive role in treatment planning also among patients who seem otherwise healthy. Such treatment decisions may impact these women’s prognosis. Our findings underline the need for specific guidelines pertaining to older patients with BC. Future studies are needed to further disentangle the impact of frailty, comorbidity, and age.

## Supplementary Information

Below is the link to the electronic supplementary material.

Supplementary file1 (PDF 864 KB)

## Data Availability

The data that support the findings of this study are not openly available due to reasons of sensitivity. Data are located in controlled access data storage at the Cancer Registry of Norway. The data used in the analyses can be made available on request to https://helsedata.no/, given the legal basis in Articles 6 and 9 of the General Data Protection Regulation (GDPR) and that the processing is in accordance with Article 5 of the GDPR. Further information is available from the corresponding author upon request.
