# Supplementary material for: Anti-HER2 treatment in everyday practice: how we treat older women with breast cancer differently
Source: Breast Cancer Res Treat. 2026 Jan 7;215(2):54. doi: 10.1007/s10549-025-07888-z (PMC12779720; doi:10.1007/s10549-025-07888-z)
Supplement: Supplementary file 1 — Supplementary file1 (PDF 864 KB) [file 10549_2025_7888_MOESM1_ESM.pdf]

Supplemental material to

Anti-HER2 treatment in everyday practice: how we treat older women with breast cancer differently

Hjorth S, Vandraas KF, Trewin-Nybråten C, Botteri E, Ursin G, Andreassen BK, Støer NC

Journal: Breast Cancer Research and Treatment

Corresponding author: Nathalie C. Støer, Department of Research, Cancer Registry of Norway, Norwegian Institute of Public Health, Oslo, Norway; nast@fhi.no

Supplementary methods

Unspecified treatments

In 2022, Sørup et al. [1], validated an algorithm for the handling of unspecified treatment codes in the Danish National Patient Registry using data from patients with different forms of cancer, but not breast cancer. Hence, we wanted to validate the algorithm for breast cancer patients, with the aim to identify the optimal upper limit for the number of days between consecutive treatments for those treatments to be considered part of the same treatment line. As data for the validation analysis was not restricted to HER2+ patients in the curative setting, we focused on chemo treatment.

For the validation, we used data from the Systemic Anti-Cancer Treatment Database (SACT), which is part of the Cancer Registry of Norway. Here, there are no unspecified treatment codes. We limited the data to treatments registered in or after 2019, as the SACT has a high level of completeness for these years. Based on these data, we first generated a gold standard for the number of chemotherapy treatment lines based on the “drug-based algorithm” from the paper by Sørup et al. [1]. We then defined the number of chemotherapy treatment lines while relying solely on the number of days between consecutive treatments. In this test, we included all treatments from SACT, as the unspecific treatment codes in NPR may represent chemotherapy, targeted therapy or other types of systemic therapy such as zoledronic acid. We tested 21 to 56 days between consecutive treatments (the 75% percentile to the 99% percentile for time between treatments from SACT). We then compared agreement, and positive predictive values for at least 1, at least 2, and at least 3 treatment lines as defined by Sørup et al. [1]. Based on this, we found the best agreement to be at either 45 or 48 days, with positive predictive values being better at 48 than at 45 days (Supplementary Figure S2). Hence, we decided to use 48 days as the upper limit for the number of days between consecutive treatments for the treatments to be considered part of the same treatment line. At 48 days, the agreement was 58.1%. The agreement in our data is expected to be much better, seeing that the validation scenario assumed that all cancer treatments from NPR were unspecified, whereas in reality, only 5.4% of the cancer treatments from NPR were unspecified.

Definition of treatment

Endocrine therapy was defined as Anatomical Therapeutic Chemical (ATC)-group L02 or ATC-group L01EF [2]. We identified trastuzumab monotherapy as ATC-code L01FD01, trastuzumab and pertuzumab combination therapy as ATC-code L01FD01 and L01FD02, or L01XY02, and trastuzumab emtansine as ATC-code L01FD03. For chemotherapies, taxanes were defined as ATC-group L01CD, anthracyclines as ATC-group, cyclophosphamide as ATC-code L01AA01, antimetabolites as ATC-group L01BC, platinum compounds as ATC-group L01XA, vinorelbine as ATC-code L01CA04, and eribulin as ATC-code L01XX41.

Adjustment strategy

We attempted to estimate the direct effect of age on anti-HER2 therapy use through adjustment and restriction on potential mediators of the age-treatment association [3]. The rationale behind this is that the total effect of age on anti-HER2 therapy use is comprised of a direct (unmediated effect) and several indirect (mediated) effects, as illustrated in Supplementary Figure S3, panel a. Adjustment (or restriction) on a mediator could introduce bias if there were unmeasured confounding of the mediator-outcome association from a covariate not affected by age (Supplementary Figure S3, panel b). However, in our case we find it more likely that the most important unmeasured covariate, functional status, is itself a mediator (Supplementary Figure S3, panel c). Thus, in our scenario, we believe that adjustment for mediators will be unlikely to introduce substantial bias. However, the "direct effect" we report will still be influenced by any unmeasured mediation.

Multiple imputation

Data were missing for the covariates household income, health region, education (each 1% missing), and tumor grade (9% missing). In addition, 1% had stage I disease and missing pathological T-staging, necessary to define whether they had indication for anti-HER2 therapy. Under the assumption that covariate data were missing at random [4], we imputed missing data using multiple imputation by chained equations [5] with 100 datasets created. The imputation models included all variables from the outcome model, as recommended [4]. The model also included hormone receptor status, and histological type as potential predictors of grade; and cardiovascular disease, diabetes, chronic obstructive pulmonary disease, and dementia (definitions described in a previous publication [6]) as potential predictors of household income. To allow for potential interactions between age group and covariates, we ran the imputation separately in each of the five age groups. Age was further included as a continuous variable in each model, seeing that the age groups were broad.

We compared results from the Poisson regression analysis on imputed data to an analysis restricted to patients with complete data on covariates and observed no substantial differences (Supplementary Table 2).

References

1. Sørup S, Darvalics B, Knudsen JS, Rasmussen AS, Hjorth CF, Vestergaard SV, Khalil AA, Russo L, Oksen D, Boutmy E, Verpillat P, Rørth M, Cronin-Fenton D (2022) Identifying Valid Algorithms for Number of Lines of Anti-Neoplastic Therapy in the Danish National Patient Registry Among Patients with Advanced Ovarian, Gastric, Renal Cell, Urothelial, and Non-Small Cell Lung Cancer Attending a Danish University Hospital. Clin Epidemiol 14:159–171. https://doi.org/10.2147/CLEP.S342238

2. WHO (2025) ATCDDD - ATC/DDD Index

3. VanderWeele TJ (2021) Mediation Analysis. In: Modern epidemiology, Fourth edition. Wolters Kluwer, Philadelphia, pp 655–675

4. Sterne JAC, White IR, Carlin JB, Spratt M, Royston P, Kenward MG, Wood AM, Carpenter JR (2009) Multiple imputation for missing data in epidemiological and clinical research: potential and pitfalls. BMJ 338:b2393

5. White IR, Royston P, Wood AM (2011) Multiple imputation using chained equations: Issues and guidance for practice. Statistics in Medicine 30:377–399. https://doi.org/10.1002/sim.4067

6. Vandraas KF, Hjorth S, Trewin-Nybråten CB, Ursin G, Botteri E, Andreassen BK, Reinertsen KV, Blix ES, Naume B, Støer NC (2025) Anti-Her2 therapy patterns in metastatic breast cancer-Real-world data suggest undertreatment. Int J Cancer. https://doi.org/10.1002/ijc.70120

Supplementary Table 1 Type of chemotherapy by age group among 3526 Norwegian women with primary non-metastatic HER2 positive breast cancer

	<55 years	55-64 years	65-74 years	75-84 years	85+ years
n	1628	824	602	324	148
Any chemotherapy	1613 (95.4%)	829 (90.8%)	594 (88.7%)	124 (34.9%)	n<5 (<3.3%)
Taxanes[a]	1577 (97.8%)	808 (97.5%)	579 (97.5%)	119 (96.0%)	n<5 (100.0%)
Anthracyclines[a]	1358 (84.2%)	656 (79.1%)	425 (71.5%)	53 (42.7%)	0
Antimetabolites[a]	697 (43.2%)	359 (43.3%)	244 (41.1%)	27 (21.8%)	0
Platinum compounds[a]	73 (4.5%)	40 (4.8%)	26 (4.4%)	6 (4.8%)	0
Vinorelbine[a]	6 (0.4%)	n<5 (<0.6%)	0	n<5 (<4.0%)	0
Eribulin[a]	n<5 (<0.3%)	n<5 (<0.6%)	n<5 (<0.8%)	0	0

[a]Proportion of those receiving any chemotherapy.

Supplementary Table 2 Numbers behind Figure 2 - treatment flows (neoadjuvant treatment, surgery and first line adjuvant treatment) by age group among 3526 Norwegian women with primary non-metastatic HER2 positive breast cancer

	<55 years	55-74 years	75+ years
n (%)	1628 (100%)	1426 (100%)	472 (100%)
Neoadjuvant anti-HER2 therapy[a]	510 (31.3%)	224 (15.7%)	55 (11.7%)
Trastuzumab[b]	263 (51.6%)	121 (54.0%)	36 (65.5%)
Trastuzumab+pertuzumab[b]	247 (48.4%)	103 (46.0%)	19 (34.5%)
Primary surgery	1097 (67.4%)	1169 (82.0%)	351 (74.4%)
Died, progressed to metastasis, were censored or reached end of follow-up[a]	21 (1.3%)	33 (2.3%)	66 (14.0%)
n followed beyond first event	1607 (98.7%)	1393 (97.7%)	406 (86.0%)
Adjuvant anti-HER2 therapy[a]	992 (61.7%)	1027 (73.7%)	123 (30.3%)
Trastuzumab[b]	917 (92.4%)	>974 (94.8%)	120 (97.6%)
Trastuzumab+pertuzumab[b]	67 (6.8%)	48 (4.7%)	<5 (<4.1%)
Trastuzumab emtansine[b]	8 (0.8%)	<5 (<0.5%)	<5 (<4.1%)
Surgery after neoadjuvant anti-HER2 therapy[a]	470 (29.2%)	197 (14.4%)	38 (9.4%)
Died, progressed to metastasis, were censored or reached end of follow-up[a]	145 (9.0%)	169 (12.1%)	245 (60.3%)
n eligible for post-neoadjuvant therapy	470 (28.9%)	197 (13.8%)	38 (8.1%)
Post-neoadjuvant anti-HER2 therapy[a]	457 (97.2%)	186 (94.4%)	32 (84.2%)
Trastuzumab[b]	274 (60.0%)	106 (57.0%)	19 (59.4%)
Trastuzumab+pertuzumab[b]	175 (38.3%)	65 (34.9%)	>8 (>25.0%)
Trastuzumab emtansine[b]	8 (1.8%)	15 (8.1%)	<5 (<15.6%)
Died, progressed to metastasis, were censored or reached end of follow-up[a]	13 (2.8%)	11 (5.6%)	6 (15.8%)

[a]Proportion of patients who were followed to this point, [b]Proportion of anti-HER2 treated

Exact numbers not shown where it could lead to identification (<5 patients in a category).

Supplementary Table 3 Association between age and use of anti-HER2 targeted therapy among Norwegian women with primary non-metastatic HER2 positive breast cancer stage I > pT1a, or II-III, diagnosed in 2015-2021, Patient Registry Index=0, and no polypharmacy, sensitivity analyses

	n	No anti-HER2 (%)	Crude RR (95% CI)	Adjusted[a] RR (95% CI)
Patients with at least 189 days of follow-up, imputed sample				
<55	877	845 (96.4)	Reference	Reference
55-64	369	350 (94.9)	0.98 (0.96-1.01)	0.99 (0.96-1.02)
65-74	199	185 (93.0)	0.96 (0.93-1.00)	0.97 (0.93-1.01)
75-84	84	59 (70.3)	0.73 (0.63-0.84)	0.73 (0.64-0.84)
85+	33	6 (18.2)	0.19 (0.09-0.39)	0.19 (0.09-0.40)
Patients with at least 56 days of follow-up and complete data on covariates				
<55	781	749 (95.9)	Reference	Reference
55-64	352	328 (93.2)	0.97 (0.94-1.00)	0.97 (0.94-1.01)
65-74	190	172 (90.5)	0.94 (0.90-0.99)	0.95 (0.90-1.00)
75-84	79	59 (74.7)	0.78 (0.68-0.89)	0.78 (0.69-0.89)
85+	31	7 (22.6)	0.24 (0.12-0.45)	0.24 (0.13-0.46)

[a]Adjusted for calendar year at diagnosis, stage, grade, household income, education, and number of hospital contacts.

RR: relative risk; CI: confidence interval.

Supplementary Figures

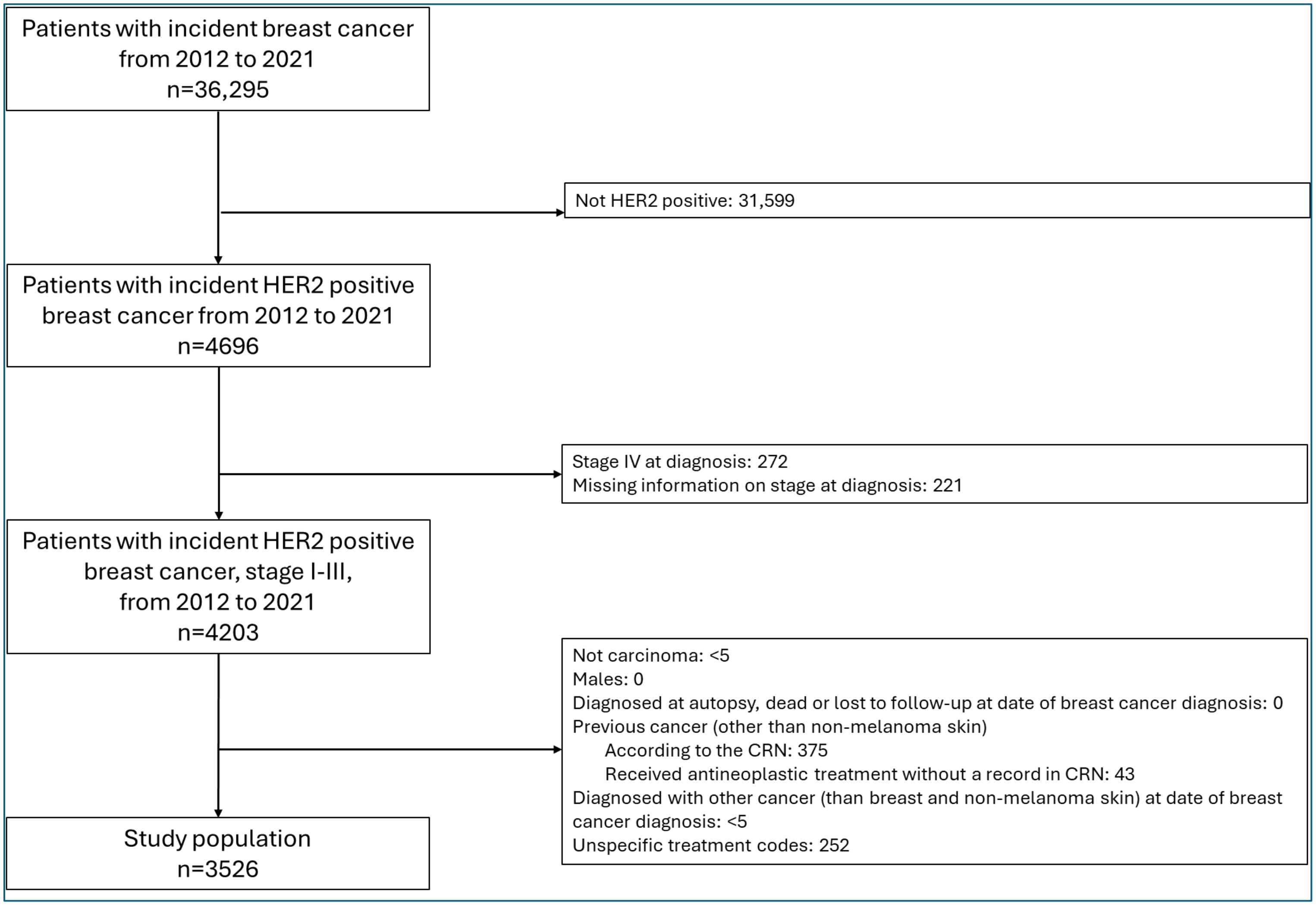

Fig. S1 Flowchart of the study population

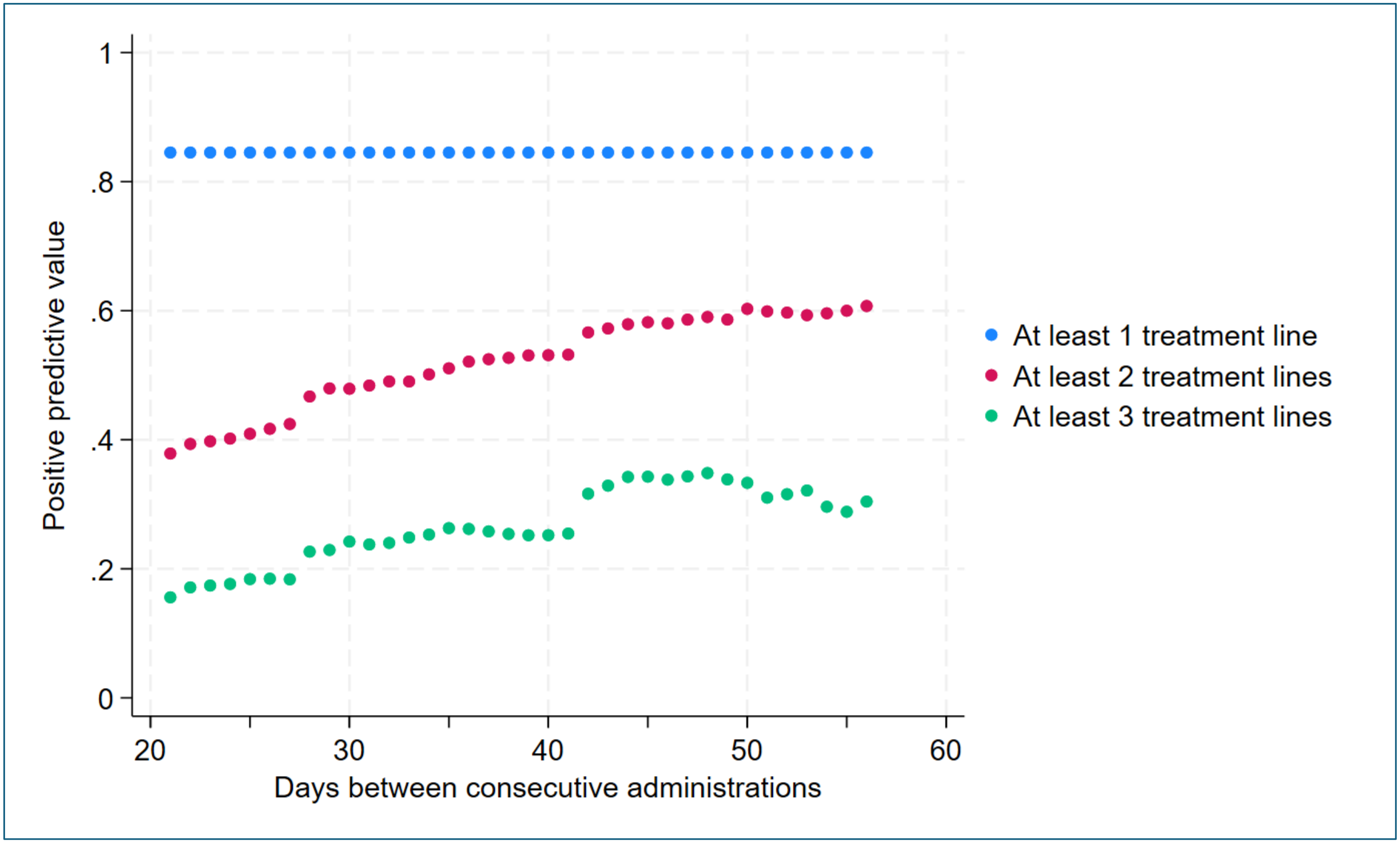

Fig. S2 Positive predictive value for number of treatment lines according to number of days between consecutive treatments, assuming that all cancer treatments from the Norwegian Patient Registry were unspecified

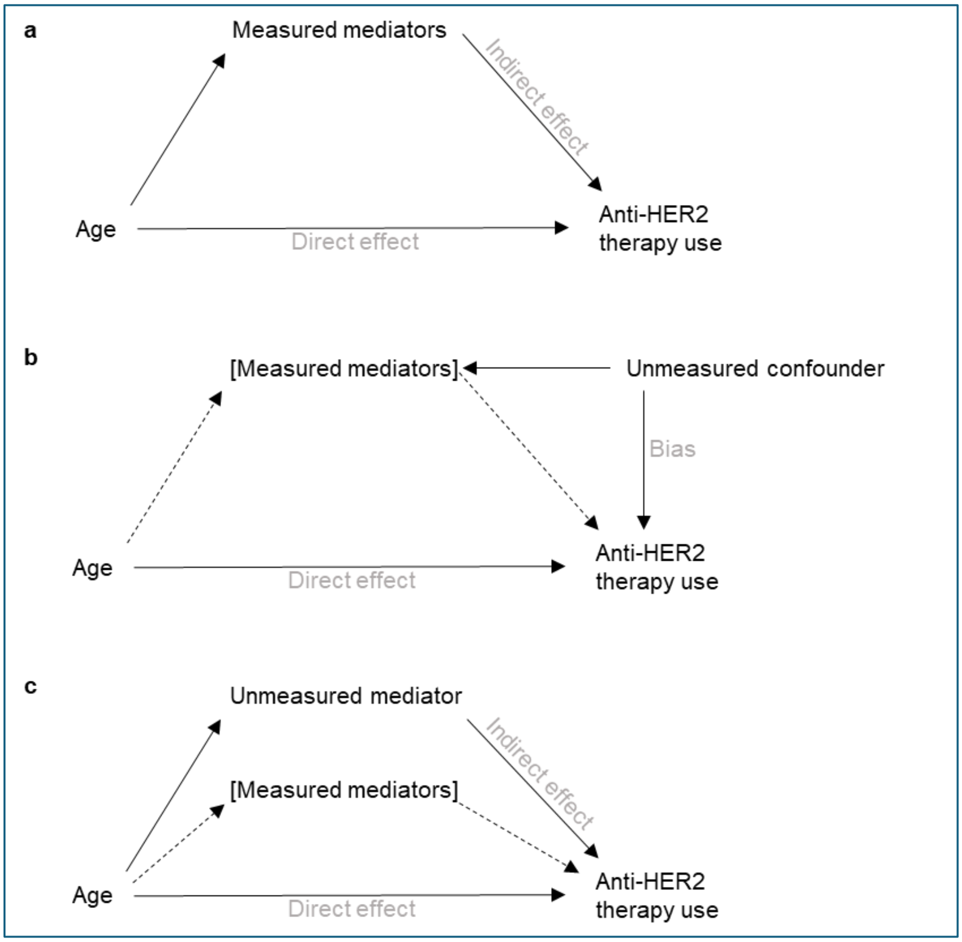

Fig. S3 Directed acyclic graphs. Panel a showing no adjustment, panel b showing adjustment under the assumption of unmeasured confounding of the mediator-outcome association, panel c showing adjustment under the assumption of unmeasured mediation, which we believe to be the most relevant scenario in our case. Measured mediators are calendar year, stage, and grade at diagnosis, household income, education, region of residence, comorbidity index, polypharmacy, and number of hospital contacts. Solid lines represent open paths, and dashed lines represent closed paths. Covariates that have been adjusted for are presented in square brackets

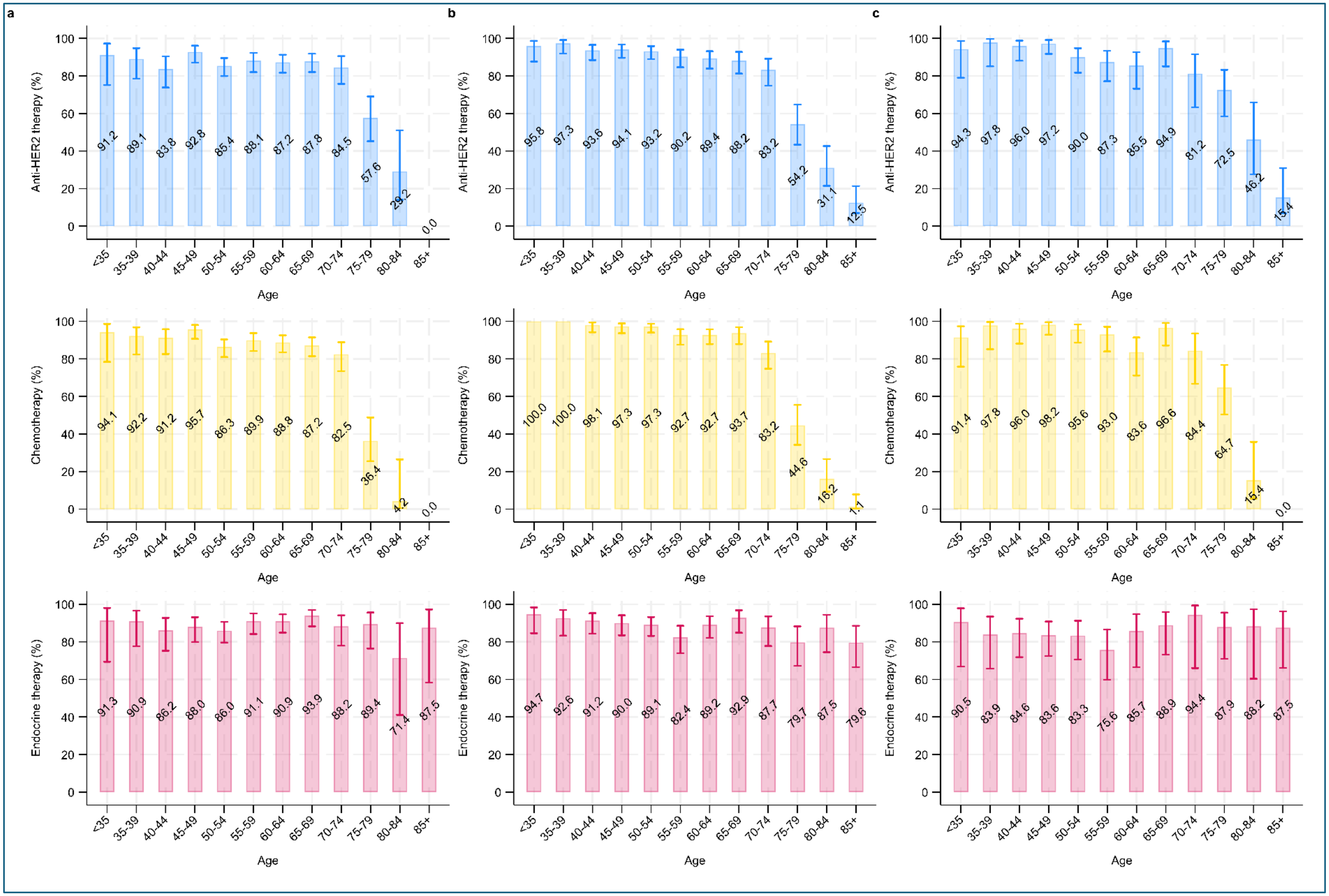

Fig. S4 Proportion of systemic treatment use by age group among 3526 Norwegian women with primary non-metastatic HER2 positive breast cancer, stratified by stage at diagnosis. Column a: stage I; column b: stage II; column c: stage III. For endocrine therapy, the denominator is patients with hormone receptor positive disease

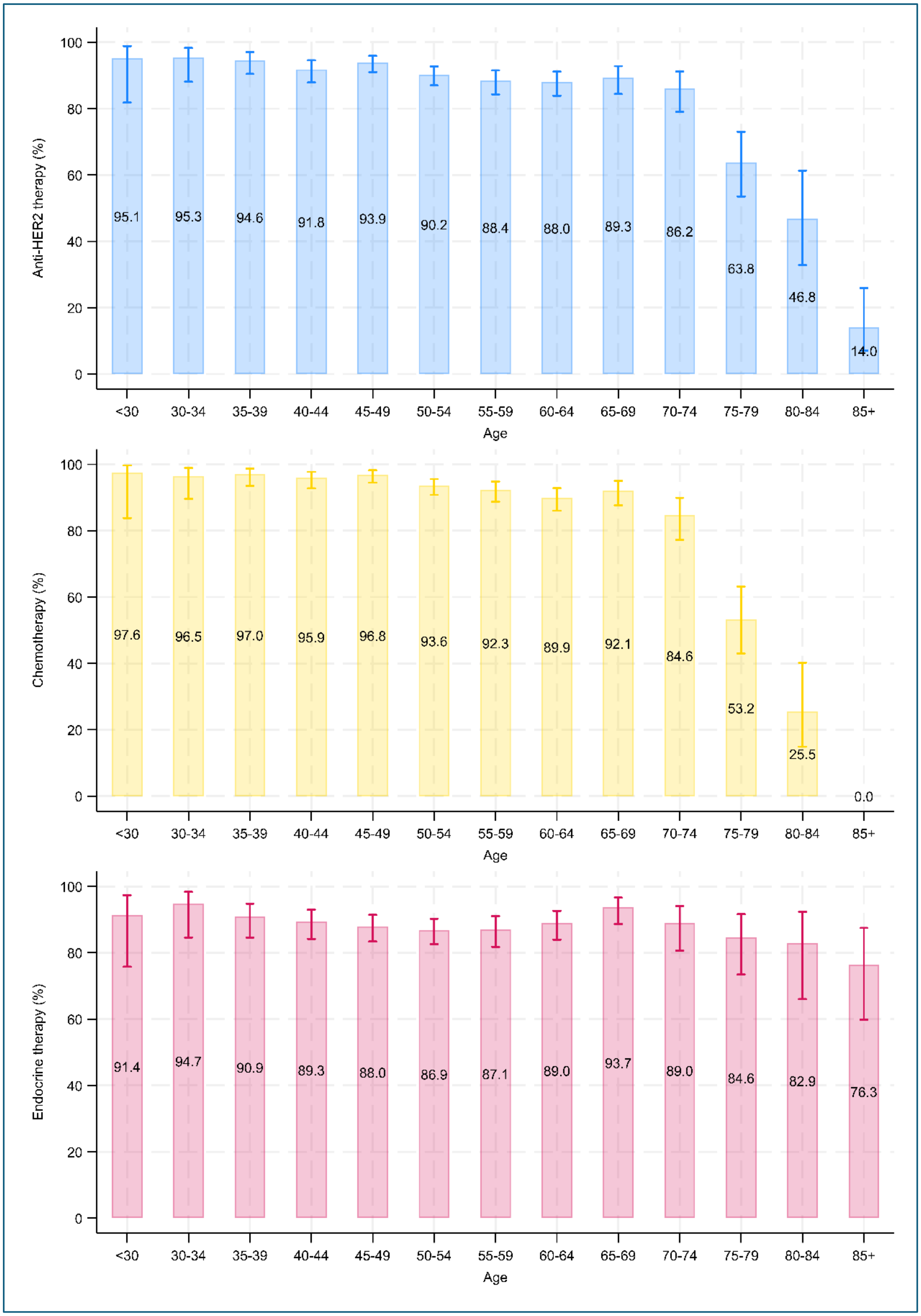

Fig. S5 Proportion of systemic treatment use by age group among 2557 Norwegian women with primary non-metastatic HER2 positive breast cancer with Patient Registry Index=0 and no polypharmacy. For endocrine therapy, the denominator is patients with hormone receptor positive disease

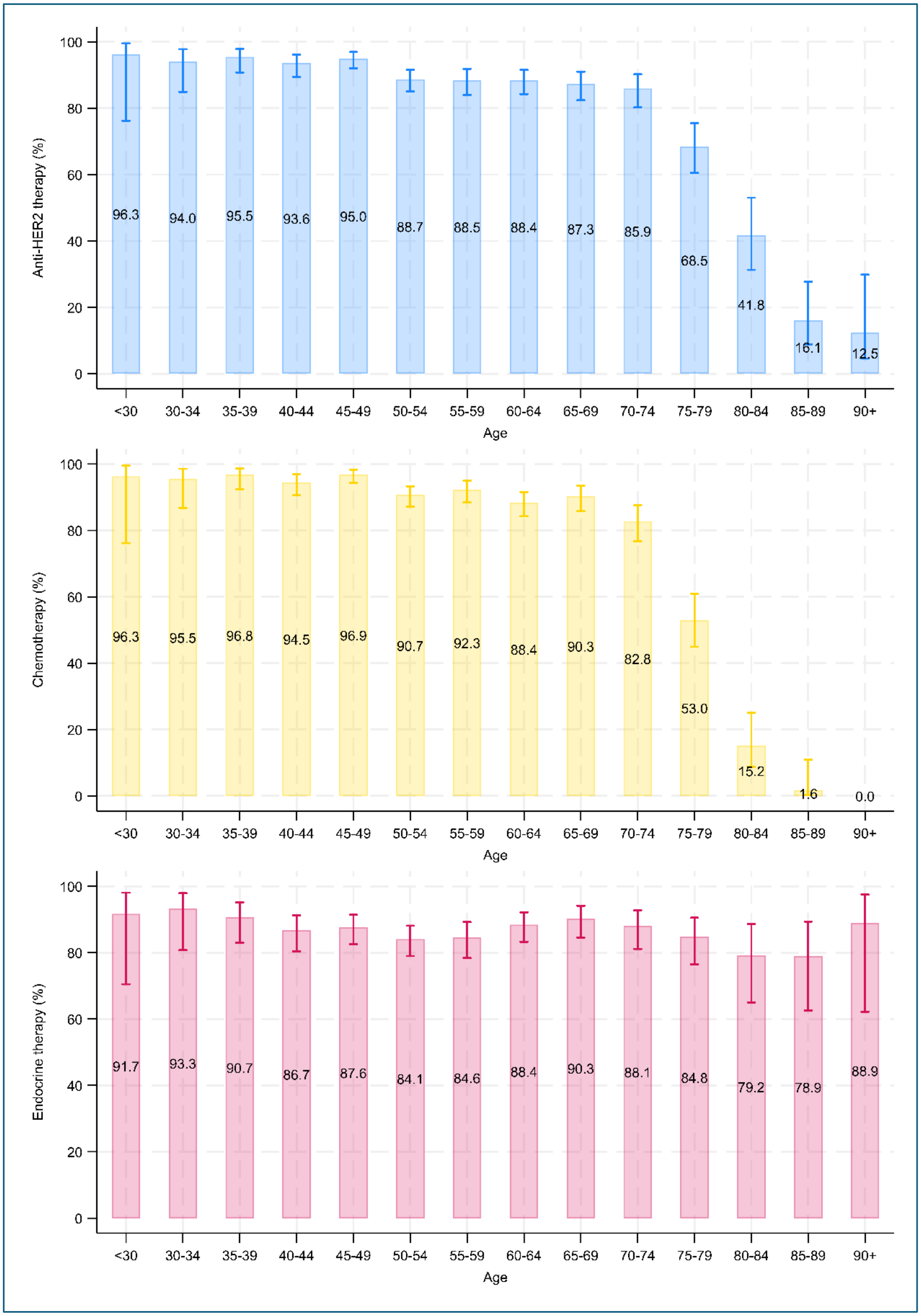

Fig. S6 Proportion of systemic treatment use by age group among 2468 Norwegian women with primary non-metastatic HER2 positive breast cancer diagnosed in 2015-2021. For endocrine therapy, the denominator is patients with hormone receptor positive disease

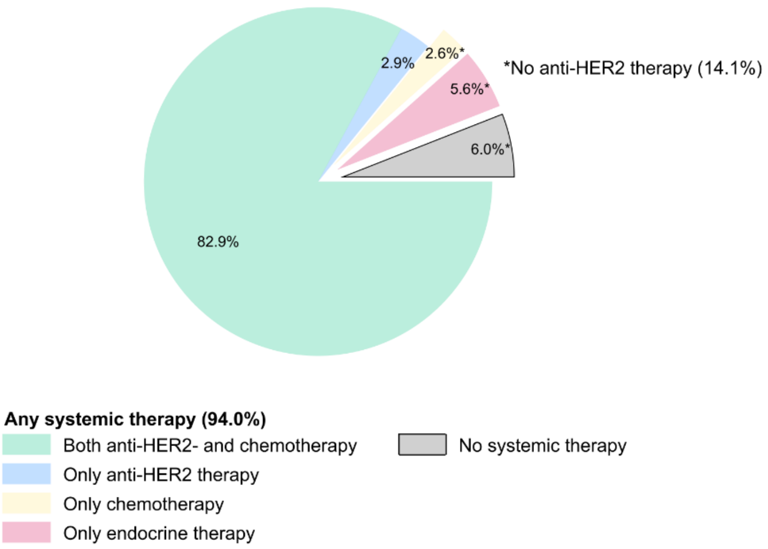

	<55 years		55-64 years		65-74 years		75-84 years		85+ years	
**Anti-HER2 treatment, n**	Yes, 1461	No, 92	Yes, 712	No, 70	Yes, 513	No, 55	Yes, 152	No, 137	Yes, 15	No, 116
Pathology stage pT1aN0M0	4.1% (3.0;5.6)	11.0% (5.5;20.6)	4.1% (2.7;6.1)	12.0% (5.4;24.6)	4.3% (2.7;6.8)	15.2% (7.3;29.1)	0	<5.1%	0	<8.1%
Either pT1aN0M0 or grade 1	5.3% (4.0;6.9)	24.6% (15.2;37.1)	5.6% (4.0;7.9)	20.5% (10.8;35.3)	7.1% (4.9;10.1)	19.6% (10.3;34.0)	<4.8%	6.2% (2.8;13.3)	0	<8.2%
Lowest income quintile at diagnosis	11.0% (9.4;12.7)	17.4% (10.9;26.7)	17.1% (14.5;20.1)	25.7% (16.7;37.4)	23.6% (20.1;27.5)	25.5% (15.5;38.8)	37.5% (30.1;45.5)	52.6% (44.1;60.8)	33.3% (13.4;61.8)	72.4% (63.5;79.8)
General health at diagnosis										
Patient Registry Index>0	4.2% (3.3;5.4)	<5.4%	7.6% (5.9;9.8)	8.6% (3.8;18.0)	17.5% (14.5;21.1)	29.1% (18.4;42.7)	20.4% (14.7;27.6)	34.3% (26.8;42.7)	<33.3%	30.2% (22.4;39.2)
Polypharmacy	11.3% (9.8;13.0)	12.0% (6.7;20.5)	20.8% (18.0;23.9)	25.7% (16.7;37.4)	34.9% (30.9;39.1)	45.5% (32.6;58.9)	46.1% (38.2;54.1)	55.5% (47.0;63.6)	46.7% (22.4;72.6)	50.0% (40.9;59.1)

Fig. S7 Characteristics of patients receiving and not receiving systemic antineoplastic treatment (Anatomical Therapeutic Chemical group L01) by age group among 3323 Norwegian women with primary non-metastatic HER2 positive breast cancer who had at least 189 days of follow-up. The percentages shown, are percentages of non-missing values. Exact percentages are not shown when <5 patients in a category
